# Prognostic significance of PD-L1 expression and tumor infiltrating lymphocyte in surgically resectable non-small cell lung cancer

**DOI:** 10.18632/oncotarget.20233

**Published:** 2017-08-12

**Authors:** Gen Lin, Xirong Fan, Weifeng Zhu, Cheng Huang, Wu Zhuang, Haipeng Xu, Xiandong Lin, Dan Hu, Yunjian Huang, Kan Jiang, Qian Miao, Chao Li

**Affiliations:** ^1^ Department of Thoracic Oncology, Fujian Cancer Hospital, Fujian Medical University Cancer Hospital, Fuzhou 350014, China; ^2^ Department of Pathology, Fujian Cancer Hospital, Fujian Medical University Cancer Hospital, Fuzhou 350014, China; ^3^ Department of Molecular Pathology, Fujian Cancer Hospital, Fujian Medical University Cancer Hospital, Fuzhou 350014, China; ^4^ Fujian Provincial Key Laboratory of Translational Cancer Medicine, Fuzhou 350014, China

**Keywords:** non-small cell lung cancer, prognosis, programmed death ligand 1, tumor infiltrating lymphocytes

## Abstract

Programmed death ligand 1 (PD-L1) expression is a predictive biomarker of the success of PD-1/PD-L1 inhibitor therapy for patients with advanced non-small cell lung cancer (NSCLC) but its role as a prognostic marker for early stage resectable NSCLC remains unclear. Here, we studied PD-L1 expression and tumor infiltrating lymphocytes (TILs) in surgically resectable NSCLC and correlate the finding with clinicopathological features and patient outcomes. Total of 170 archival samples of resectable NSCLC were probed for PD-L1 expression using the clone 22C3 pharmDx kit. The PD-L1 expression was determined by the Tumor Proportion Score (TPS) and classified into TPS <1%, TPS 1 to 49% and TPS ≥50%. The scoring of TILs was from hematoxylin & eosin stained tissue sections using a system for standardized evaluation of TILs in breast cancer. PD-L1 expression was compared with clinical pathological characteristics and survival outcome. Expression of PD-L1 scores of TPS ≥50%, TPS 1 to 49% and TPS <1% were observed in 10.6%, 24.7% and 64.7% of the 170 archival samples, respectively. Positive PD-L1 expression was significantly higher in patients with squamous carcinoma, in those with higher TNM stage and with the presence of TILs. Neither the PD-L1 expression, TIL status, nor their combination was an independent prognosis biomarker of survival when the data was subjected to either univariate or multivariate analysis. The incidence of PDL1 expression appears to be lower in patient with early stage resectable lung cancer. PD-L1 expression and TILs are not prognostic indicators of survival outcome in this population.

## INTRODUCTION

Blockade of immune checkpoints has recently emerged as a novel treatment for various cancers. In particular, monoclonal antibodies targeting programmed cell death 1 (PD-1) or its ligand (PD-L1) have been extensively studied in lung cancer, and their roles as first-line [1] or second-line [2–6] treatment in the management of advanced non-small cell lung cancer (NSCLC) patients are well established. The reports from high-profile clinical trials have shown an association of PD-L1 expression determined by immunohistochemistry (IHC) with overall response rates which suggested that PD-L1 expression may be a clinically applicable predictive biomarker [1, 2, 7].

The recent clinical series had reported various degrees of PD-L1 expression in lung cancers ranging from 7.4% to 72.7%, which was well reviewed by Mino-Kenudson [8]. The same series have correlated PD-L1 expression with clinicopathologic characteristics, molecular variables, and survival but the results vary greatly [8–13]. Each PD-1 or PD-L1 inhibitor is developed with a companion diagnostic biomarker using a different PD-L1 antibody (clones SP263, SP142, 22C3, 28-8, and etc.), IHC platforms, and scoring systems, which make a head-to-head comparison between studies difficult. Among the PD-L1 antibodies, only the PD-L1 IHC 22C3 pharmDx assay has obtained regulatory approval as a companion diagnostic, which is linked to the use of pembrolizumab [1, 2, 7]. Clinical application of the 22C3-PD-L1 biomarker was essentially confined to patients with advanced NSCLC and there is only limited information on patients with early stage resectable lung cancer. Recently, commercial 22C3-PD-L1 IHC diagnostic assays became available. However, to our knowledge, the clinical data linked to clinicopathologic factors and survival are very limited and the results also inconsistent, even using the FDA approved standard 22C3 PD-L1 antibody [12, 14, 15].

Primary mechanism of PD-L1 on tumors is innate immune resistance and adaptive immune resistance. Interferon gamma (IFN-γ) is released by CD8+ T-cells and is a major inducer of PD-L1 expression *in vivo* [16, 17]. In addition, PD-L1 expression was up-regulated secondary to constitutive oncogenic signaling within tumor cells, which is evidenced by the small fraction of human cancers that lack tumor infiltrating lymphocytes (TILs) in the tumor microenvironment but still express high levels of PD-L1 [11, 16, 18–22]. Recently, multiple studies of various malignancies have shown that PD-L1 expression is associated with significant TIL infiltration of the tumor microenvironment. However, a standardized methodology for evaluating TILs in lung cancer is still unavailable and several studies with NSCLC cohorts have investigated TILs in association with PD-L1 expression, again, producing conflicting results [8].

To address the issue, we investigate association with clinicopathologic characteristics and the prognostic value of PD-L1 expression measured by a commercial 22C3-PD-L1 immunohistochemistry diagnostic assay with a Dako platform in patients with surgically resectable NSCLC. We have also explored the immune microenvironment by studying the association between PD-L1 expression and tumor lymphocyte infiltration.

## RESULTS

### Patient characteristics

A total of 170 patients were eligible for study and their characteristics are summarized in Table 1. Median age at diagnosis was 56 years (range, 34-78 years) and 118/170 (69.4%) patients were male. The ECOG performance status was 0 in all patients. Adenocarcinoma and squamous carcinoma accounted for 94/170 (55.3%) and 76/170 (44.7%), respectively. Fifty patients (29.4%), 43 (25.3%), and 77 (45.3%) had stage I, stage II and stage III disease, respectively (Table 1). The EGFR and ALK status were not routinely detected in China between 2008 and 2010 and data is not always available in the medical records. As a result, only 13 patients had their EGFR status known (10 EGFR mutations, 5 wild-type), and 13 patients had known ALK status (12 negative, 1 positive). Ninety-six of 120 (80.0%) patients with stage II and stage III disease have been offered adjuvant chemotherapy.

**Table 1:** Association between 22C3-PD-L1 protein expression and clinicopathological factors

Subgroup		*N*	PD-L1 expression N(%)			Univariate analysis	Multivariate analysis
			TPS <1%	1 to 49%	≥50%	OR(95%CI) *P* value	OR(95%CI) *P* value
**Overall**		170	110(64.7%)	42(24.7%)	18(10.6%)		
**Gender**							
	**Female**	52	38(73.1%)	12(23.1%)	2(3.8%)	1.73 (0.85-3.55)	
	**Male**	118	72(61.0%)	30(25.4%)	16(13.6%)	0.132	
**Age**							
	**≤60y**	99	63(63.6%)	27(27.3%)	9(9.1%)	0.89 (0.47-1.70)	
	**>60y**	71	47(66.2%)	15(21.1%)	9(9.3%)	0.730	
**Smoking status**							
	**Never-smoke**	97	68(70.1%)	20(20.6%)	9(9.3%)	1.73 (0.92-3.27)	
	**Smokers**	73	42(57.5%)	22(30.1%)	9(12.3%)	0.091	
**Histology**							
	**AD**	94	69(73.4%)	19(20.2%)	6(6.4%)	2.36 (1.24-4.48)	2.02 (1.01-4.01)
	**SCC**	76	41(53.9%)	23(30.3%)	12(15.8%)	**0.009**	0.045
**Tumor location**							
	**Peripheral**	74	53(71.6%)	16(21.6%)	5(6.8%)	1.73 (0.90-3.31)	
	**Central**	96	57(59.4%)	26(27.1%)	13(13.5%)	0.099	
**TNM stage**							
	**I**	50	40(80.0%)	7(14.0%)	3(6.0%)	Reference	Reference
	**II**	43	25(58.1%)	8(18.6%)	10(23.3%)	2.88 (1.15-7.23)	2.68 (1.03-7.02)
	**III**	77	45(58.4%)	27(35.1%)	5(6.5%)	2.84 (1.24-6.51)	3.53 (1.48-8.42)
						**0.031**	0.016
**TILs**							
	**Absence**	29	25(86.2%)	4(13.8%)	0(0.0%)	4.12 (1.36-12.47)	5.32 (1.69-16.68)
	**Presence**	141	85(60.3%)	38(27.0%)	18(12.8%)	**0.012**	**0.004**

AD, adenocarcinoma; SCC, squamous carcinoma.

### PD-L1 expression and correlation with clinicopathological characteristics

**Figure 1:**
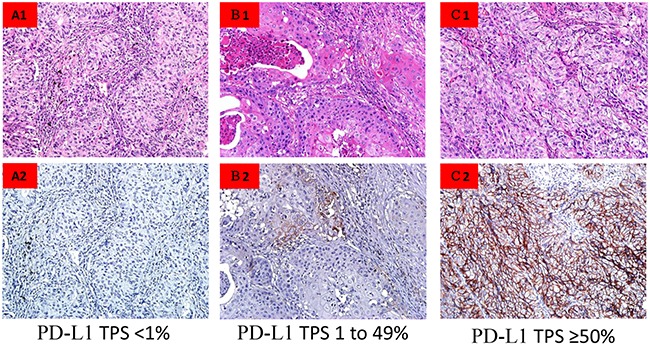
PD-L1 immunohistocehmistry labeling in NSCLC tumor specimens **(A)** PD-L1 TPS <1%. **(B)** PD-L1 TPS 1 to 49%. **(C)** PD-L1 TPS ≥50%.

PD-L1 was commonly expressed at the cell membrane of cancer cells, and only in selective cases in the cytoplasm. Heterogeneous distribution of PD-L1 staining was observed within a single section of tumor tissue, with some areas being dominated by cells with strong PD-L1 expression, whereas other areas were characterized by cells lacking PD-L1 expression. Representative examples of PD-L1 Tumor Proportion Score (TPS) <1%, TPS 1 to 49%, and TPS ≥50% are shown in Figure 1. The PD-L1 TPS ≥50% and TPS 1 to 49% were observed in 10.6% and 24.7% of patient tumors. Among the 60 cases that were considered PD-L1 positive (TPS ≥1%), the median percentage of tumor cells with positive staining was 30% (interquartile range, 2%-50%).

Expression of PD-L1 was correlated with the clinicopathological characteristics by univariate analysis in a two-level classification of tumors that were negative (TPS <1%) vs. positive (TPS ≥1%) for PD-L1 expression (Table 1). There was no statistically significant association between PD-L1 expression and gender, age, smoking status and primary tumor location in the univariate analysis (Table 1).

Histology is strongly correlated with PD-L1 expression. Incidence of positive PD-L1 expression in squamous carcinoma tumors was 46.1% comparing to 26.6% in adenocarcinoma tumors (OR 2.36; 95%CI, 1.24-4.48, *p*=0.009). In this study, higher tumor stage was significantly associated with PD-L1 expression in univariate analysis, stage I 20.0%, stage II 41.9%, and stage III 41.6%, *p*=0.031. In the multivariate binary logistic analysis, squamous carcinoma tumors and higher TNM stage was confirmed as significantly independent factors for higher incidence of PD-L1 expression (Table 1).

### TILs and classification of tumor immune microenvironment

**Figure 2:**
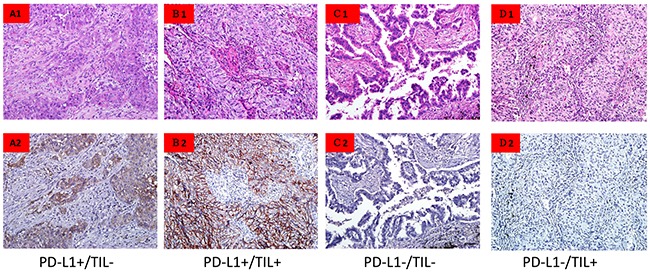
Tumor cell PD-L1 expression and lymphocytic infiltration **(A)** PD-L1+TILs-. **(B)** PD-L1+TILs+. **(C)** PD-L1-TILs-. **(D)** PD-L1-TILs+. TILs showed PD-L1 positive expression in (D). 20X.

TILs were observed in 141 (82.9%) of the tumors (Table 1). Representative examples of different types of tumor immune microenvironments are shown in Figure 2. In the present study, we found 56 (39.8%) PD-L1 positive tumors and 85 (60.3%) PD-L1 negative tumors among the tumors with TILs present. The tumors without TILs included four (13.8%) tumors that were PD-L1 positive and 25 tumors (86.2%) that were PD-L1 negative. Furthermore, there were no cases of the subtype of PD-L1 TPS ≥50% detected in the tumors without TILs. The odds ratio (OR) was 4.12 (95% CI, 1.36-12.47), *p*=0.012, by univariate analysis and OR 5.32 (95% CI, 1.69-16.68), *p*=0.004, by multivariate analysis (Table 1).

### Prognostic value of PD-L1 expression and TILs

Survival data in this study were censored on January 07, 2017. The median follow-up time was 53.8 months (m) (range: 1.4 to 104 m) and 89 patients had cancer-related deaths.

**Figure 3:**
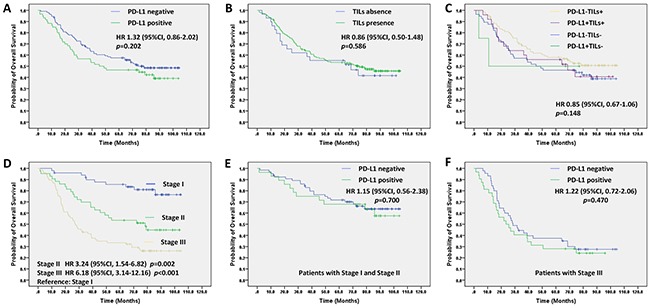
Prognostic value of PD-L1 expression and TILs status and interaction between them in the univariate survival analysis **(A)** for PD-L1 expression and OS. **(B)** for TILs status and OS. **(C)** for interaction between PD-L1 and TILs status and OS. **(D)** for TNM stage and OS. **(E)** for PD-L1 expression in patients with Stage I and stage II and OS. **(F)** for PD-L1 expression in patients with Stage III and OS.

In the univariate survival analysis the PD-L1 negative expression group had a tendency to have a longer overall survival than the PD-L1 positive expression group, median overall survival 67.6 months (95%CI, 60.2-74.9) vs. 57.9 months (95%CI, 47.4-68.4), HR 1.32 (95%CI, 0.86-2.02), *p*=0.202 (Figure 3A). Using the TNM stage as a stratification factor (Stage III vs. Stage I+II), PD-L1 expression was still not associated with overall survival in the population (Figure 3E, 3F). Similarly, there was no significant difference in OS between patients with TILs and without TILs, HR 0.86; 95%CI, 0.50-1.48; *p*=0.586 (Figure 3B). Furthermore, TIL status was not an independent prognostic factor for overall survival using 50% stromal TILs as the cut-off (Table 2). The patients were divided into four subgroups; PD-L1+TILs+, PD-L1+TILs-, PD-L1-TILs+ and PD-L1-TILs-. Kaplan-Meier graphical analysis demonstrated that OS was not significantly different among the four subgroups, HR 0.85; 95%CI, 0.67-1.06, *p*=0.148 (Figure 3C). The unadjusted survival curves show a statistically significant association between TNM stage and survival, which also confirmed in the multivariate Cox regression analysis after controlling for covariates (Figure 3D; Table 2).

**Table 2:** Univariate and multivariate analyses of OS in all patients using the 1% cutoff value

Variables	Reference	Univariate analyses		Multivariate analyses	
		HR(95% CI)	*P value*	HR(95% CI)	*P value*
**Gender**	**Male**	1.12 (0.71-1.77)	0.626		
**Age**	**≤60y**	1.20 (0.79-1.83)	0.391		
**Histology**	**Squamous**	1.05 (0.69-1.59)	0.83		
**Smoking status**	**Never smokers**	1.26 (0.83-1.90)	0.286		
**TNM stage**	**I (reference)**		**<0.001**		**<0.001**
	**II**	3.24 (1.54-6.82)	**0.002**	3.58 (1.69-7.56)	**0.001**
	**III**	6.18 (3.14-12.16)	**<0.001**	5.11(2.49-10.48)	**<0.001**
**PD-L1 expression**	**Negative**	1.32 (0.86-2.02)	0.202		
**PD-L1 expression in Stage I and II**	**Negative**	1.15 (0.56-2.38)	0.700		
**PD-L1 expression in Stage III**	**Negative**	1.22 (0.72-2.06)	0.470		
**TILs**	**Absence**	0.86 (0.50-1.48)	0.586		
**TILs**	**>50% TILs**	0.71 (0.43-1.18)	0.189		
PD-L1 expression^*^ TILs		0.85 (0.67-1.06)	0.148		

## DISCUSSION

We studied PD-L1 expression and TILs in 170 patients with resectable NSCLC and found neither the PD-L1 expression, TILs status, nor their combination to be an independent prognosis biomarker. The incidence of positive PDL1 expression is lower than expected. This could be related to the early stage of disease.

We noticed that many clinical series of lung cancers had reported various amounts of PD-L1 expression and correlate expression with clinicopathologic characteristics and/or survival leading to conflicting results [8]. One of interpretation of the discrepancy could be different antibodies, platforms, and cut-off values when comparing the experimental positives used in the different studies. Hirsch et al. studied the concordance between four PD-L1 antibodies (SP142, SP263, 22C3 and 28-8) and found similar analytical performance for three assays (SP263, 22C3 and 28-8). There were cases of misclassification of PD-L1 status comparing SP142 to the other three [23, 24]. Echoing Hirsch, another study compared the performance of four PD-L1 platforms, including the 28-8 antibody on the Dako Link 48 platform, the 22c3 antibody on the Dako Link 48 platform, the SP142 antibody on the Ventana Benchmark platform, and the E1L3N antibody on the Leica Bond platform. Results showed that the assay using the SP142 antibody was an outlier that detected significantly less PD-L1 expression in tumor cells and immune cells. The assay using antibody 22C3 showed slightly yet significantly less staining than either 28-8 or E1L3N [25].

Among the above PD-L1 antibodies, it is only the PD-L1 IHC 22C3 pharmDx assay that has obtained regulatory status as a companion diagnostic. We performed a literature search in PubMed for studies using the 22C3-PD-L1 assay published before Mar 1, 2017 [1, 2, 7, 12, 14, 15, 26] that had an association with clinicopathologic characteristics and/or survival (summarized in Table 3). The prevalence of 22C3-PD-L1 positive expression appears to be different between advanced NSCLC and early stage NSCLC. Three clinical trials (KEYNOTE-001, KEYNOTE-010 and KEYNOTE-024) have shown that the TPS ≥50% in patients with advanced NSCLC was between 23.2% and 30.2% and TPS (1 to 49%) was reported in 37.6% to 37.9% [1, 2, 7]. We found a lower incidence of positive PD-L1 expression in our cohort of patients with early stage NSCLC. Furthermore, our study showed that the PD-L1 expression is more likely influenced by disease stage, which indicated that the induction of PD-L1 expression was not an initial event in the development of cancer. A recent study echoes our finding, which report the incidence TPS ≥50% at 7.4% and TPS 1-49% at 20.8% [12]. We also found squamous carcinoma tumors to be strongly associated with PD-L1 expression. The biological determinants and potential clinical implications of these observations are unknown and require further study.

**Table 3:** Summary of recent studies investigating 22C3-PD-L1 expression in NSCLC

Author	N	PD-L1 (%)	Histology (%)	Stage (%)	Correlation withPD-L1 positive	Prognosis ofPD-L1 positive
		1-49%/ >50%	ADs/Sq/others	I/II/III/IV		
Reck M^1^	1653	-/30.2	-	0/0/0/100	-	-
Herbst RS^2^	2222	37.9/28.5	-	0/0/0/100	-	-
Garon EB^7^	824	37.6/23.2	81.0^*^/17.2/1.8	0/0/0/100	KRAS mutation	-
Cooper WA^12^	678	20.8/7.4	40.7/40.0/19.3	50/50(II+III)/0	Younger: High tumor grade	Better
He Y^14^	139	-/18.0	28.8/58.3/12.9	41.7/25.2/28.1/5.0	NS	Poor
Sorensen SF^15^	204	50/25	72/21.6/6.4	-/-/-/88	NS	NS
Rangachari D^26^	71	28.2/29.6	100/0/0	25.4(I-III)/74.6	Smoking	-
Present study	170	24.7/10.6	55.3/44.7	29.4/25.3/45.3/0	Histology; TILs; tumor stage	NS

-, data not shown; NS, not significant; ^*^, nonsquamous.

Recent studies, including ours, have investigated the prognostic impact of PD-L1 expression in NSCLC. However, the results are conflicting [8]. Differences in PD-L1 antibody clones used in the various studies could contribute to the conflicting results. In fact, the results were inconsistent even in three studies using the same 22C3 PD-L1 antibody [12, 14, 15]. One study found high PD-L1 expression was associated with early postoperative recurrence in a Korean population of early and advanced stage NSCLC, particularly in adenocarcinoma [14]. In contrast, PD-L1 high expression appears to be a favorable prognostic factor in a cohort of 678 patients with early stage disease [12]. In another study, PD-L1 expression is not a strong prognostic indicator in a European population of patients with advanced stage NSCLC treated with chemotherapy [15], which was similar to our results. Based on our study, PD-L1 expression is more likely influenced by tumor stage, therefore, the dynamics of PD-L1 expression may also limit its use as a prognostic biomarker.

A framework was previously proposed to stratify the tumor microenvironment into different types based on the presence or absence of TILs and PD-L1 expression [16, 27, 28]. We observed TILs presence in most patients with positive PD-L1 expression. All patients with PD-L1 TPS ≥50% were found to have presence of TILs. Brambilla et al. found intense TILs (>50% stromal TILs) in a minority of tumors that was a favorable prognostic marker for survival in resected non-small-cell lung cancer [9]. However, our analysis showed the contrary. One of reason is that pathologists have shown poor agreement of the scoring of immune cells probed with different antibodies [25]. In addition, it is not surprising using that TILs as a single factor has been found to produce paradoxical results for survival. The role of TILs in cancer growth is complex, and TILs may both promote or suppress tumor progression [29]. For the immediate future, it is critical to focus on a specific cell subsets within TILs and delve deeply into the details of the TILs present and to characterize them in relation to genetic and microenvironment characteristics.

In conclusion, incidence of PD-L1 expression (by 22C3) in patients with resectable NSCLC is relatively lower than in patients with advanced NSCLC. Our findings do not support PD-L1 expression, TILs, or the combination of both as a significant prognostic factor for resectable NSCLC.

## MATERIALS AND METHODS

### Patients and materials

Primary tumor samples were from the archive of patients with surgically resectable NSCLC and pathologically confirmed adenocarcinoma or squamous cell carcinoma at the Fujian Cancer Hospital in China between January 2008 and December 2010. None of the patients had prior anti-PD-1/PD-L1 therapies, neo-adjuvant chemotherapy or EGFR/ALK-targeted therapy. The clinicopathologic information of patients was collected from the clinical records and pathology reports. The pathological TNM stage was reassigned according to the 8^th^ TNM staging [30] and lung tumor histology were reclassified according to the 2015 World Health Organization (WHO) classification for lung tumors [31]. Patients with stage II/III disease may have been offered adjuvant chemotherapy and patients with recurrent disease received chemotherapy and/or EGFR-targeted therapy. The study design was approved by the Ethical Committee of Fujian Cancer Hospital; and written informed consent was obtained from all patients.

### PD-L1 immunohistochemistry

We conduct the study on PD-L1 expression at the Chinese University of Hong Kong, using automated staining by the Autostainer Link 48 with the murine 22C3 anti-human PD-L1 antibody (Code SK006, Merck & Co., Inc., Hong Kong) according to the manufacturer's protocol. Each staining run contained positive and negative controls along with a negative isotype-matched antibody control for each sample. Two board-certified pathologists (CL, DH) independently evaluated all stained slides for PD-L1 membrane staining. All areas in a tissue section were observed to appropriately evaluate the expression of PD-L1 on tumor cells. Following the standard recommendation as per prior publications, PD-L1 expression was determined by the TPS and classified into TPS <1%, TPS 1 to 49% and TPS ≥50%. [1, 2, 7]. Using PD-L1 TPS ≥1% as the cut off, PD-L1 expression were classified into positive (TPS ≥1%) and negative (TPS <1%) groups.

### Evaluation of tumor infiltrating lymphocytes

The independent scoring of TILs was performed in hematoxylin & eosin stained formalin fixed paraffin embedded tissue sections by two pathologists (CL, DH). Due to the lack of a standardized methodology for evaluating TILs in lung cancer, we adopted a recently reported system for standardized evaluation of TILs in breast cancer, which is also adopted in lung cancer [32]. Briefly, TILs were classified into three groups as no/minimal immune cells (0-10% stromal TILs), intermediate/heterogeneous infiltrates (10-40% stromal TILs), and high immune infiltrates (40-90% stromal TILs). The agreement analysis showed that the kappa varied from 0.40 to 0.76 for three groups and from 0.67 to 0.86 for two groups, TILs absent vs. TILs present with >10% stromal TILs as the cut off. The discordant cases were reviewed to reach a final consensus classification. After the concordance analysis, the lymphocyte infiltration was considered as a binary marker (TILs absent vs. TILs present) for the statistical analysis.

### Classification of tumor immune microenvironment

Based on PD-L1 expression and TILs status, we classified the tumor immune microenvironment into four categories: 1) PD-L1 positive expression and TILs present (PD-L1+TILs+), 2) PD-L1 positive expression and TILs absent (PD-L1+TILs-), 3) PD-L1 negative and TILs present (PD-L1-TILs+), 4) PD-L1 negative and TILs absent (PD-L1-TILs-).

### Statistical analysis

Using PD-L1 TPS ≥1% as the cut off, PD-L1 expression were classified into positive (TPS ≥1%) and negative (TPS <1%) groups. The level of PD-L1 positive expression was compared in subgroups based on age (≤60yr or >60yr), gender (male or female), smoking status (never smoker or former/current smoker), primary tumor location (central or peripheral), histology (adenocarcinoma or squamous carcinoma), TNM stage (I, II, or III) and TILs (absence or presence) using the binary logistic analysis. Adjustment was made for age, gender, smoking status, primary tumor location, histology, TNM stage and TILs in multivariate binary logistic analysis.

Overall survival (OS) was defined as the time from the date of diagnosis to the date of death or the last follow-up. The Kaplan-Meier method and a log-rank test were used for univariate survival analysis. Survival rate correlation of PD-L1 expression with, age, gender, smoking status, histology, TNM stage, PD-L1 expression, TILs and interaction between PD-L1 expression and TILs were estimated by the Kaplan-Meier method and survival curves were compared with the log-rank test. Cox proportional hazard models were used for multivariate survival analysis that controlled for the above factors and the hazard ratio (HR) and 95% CI were estimated.

Statistical analyses were performed using SPSS16.0 software. All tests were two-sided. Statistical significance was set at *p*<0.05.

## Abbreviations

PD: L1: programmed death ligand 1
NSCLC: non-small cell lung cancer
TILs: tumor infiltrating lymphocytes
TPS: Tumor Proportion Score
PD-1: programmed cell death 1
IHC: immunohistochemistry
IFN-γ: interferon gamma
AD: adenocarcinoma
SCC: squamous carcinoma
NS: not significant
WHO: World Health Organization
OS: overall survival
HR: hazard ratio
FDA: Food and Drug Administration
CI: confidence interval
